# Involvement of insulin resistance in D-galactose-induced age-related dementia in rats: Protective role of metformin and saxagliptin

**DOI:** 10.1371/journal.pone.0183565

**Published:** 2017-08-23

**Authors:** Sara Kenawy, Rehab Hegazy, Azza Hassan, Siham El-Shenawy, Nawal Gomaa, Hala Zaki, Amina Attia

**Affiliations:** 1 Pharmacology Department, Medical division, National Research Centre, Giza, Egypt; 2 Pathology Department, Faculty of Veterinary Medicine, Cairo University, Giza, Egypt; 3 Pharmacology and Toxicology Department, Faculty of Pharmacy, Cairo University, Cairo, Egypt; Nathan S Kline Institute, UNITED STATES

## Abstract

Age-related dementia is one of the most devastating disorders affecting the elderly. Recently, emerging data suggest that impaired insulin signaling is the major contributor in the development of Alzheimer’s dementia (AD), which is the most common type of senile dementia. In the present study, we investigated the potential therapeutic effects of metformin (Met) and saxagliptin (Saxa), as insulin sensitizing agents, in a rat model of brain aging and AD using D-galactose (D-gal, 150 mg/kg/day, s.c. for 90 successive days). Six groups of adult male Wistar rats were used: normal, D-gal, Met (500 mg/kg/day, p.o), and Saxa (1 mg/kg/day, p.o) control groups, as well as D-gal/Met and D-gal/Sax treated groups. Impaired learning and memory function was observed in rats treated with D-gal using Morris water maze test. Biochemical and histopathological findings also revealed some characteristic changes of AD in the brain that include the increased content of acetylcholine, glutamate, and phosphorelated tau, as well as deposition of amyloid plaques and neurofibrillary tangles. Induction of insulin resistance in experimentally aged rats was evidenced by increased blood glycated hemoglobin, brain contents of insulin and receptors for advanced glycated end-products, as well as decreased brain insulin receptor level. Elevation of oxidative stress markers and TNF-α brain content was also demonstrated. Met and Saxa, with a preference to Met, restored the normal memory and learning functions in rats, improved D-gal-induced state of insulin resistance, oxidative stress and inflammation, and ameliorated the AD biochemical and histopathological alterations in brain tissues. Our findings suggest that D-gal model of aging results in a diminishing of learning and memory function by producing a state of impaired insulin signaling that causes a cascade of deleterious events like oxidative stress, inflammation, and tau hyper-phosphorylation. Reversing of these harmful effects by the use of insulin-sensitizing drugs like Met and Saxa suggests their involvement in alleviation insulin resistance as the underlying pathology of AD and hence their potential use as anti-dementia drugs.

## Introduction

Aging and geriatric disorders are becoming one of the major concerns of the current medicine as the world's population is aging at a fast rate probably due to the advances in science and medicine [1]. Dementia is one of the most devastating geriatric disorders and characterized by a deterioration of intellectual functions such as cognition, memory, and judgment. Higher cortical functions such as language, reasoning, and the ability to follow directions are also impaired. Of all the dementias, Alzheimer’s dementia (AD) is the most common; it contributes to 60–75% of all dementia cases [2]. The pathological and histological hallmarks of AD include amyloid plaques and neurofibrillary tangles [3, 4].

Emerging data demonstrate that there is a strong link between AD and type 2 diabetes mellitus (DM). The pathogenesis of the latter involves a progressive development of insulin resistance leading to hyperglycemia. Several studies have shown that in the early stages of AD cerebral glucose utilization and blood flow is reduced by as much as 45% and 18%, respectively. Therefore, altered brain metabolism with features resembling DM is detectable soon after the onset of dementia-related symptoms [5]. Analysis of postmortem human brains has also demonstrated that these abnormalities correlate to the severity of dementia and neurodegeneration [6]. In light of the previous data, this relation between AD and DM led to a hypothesis, namely the type 3 hypotheses, which assumes that a brain-form of type 2 DM is the major contributor to the development of neurodegeneration and AD pathology instead of being its consequence. Accordingly, in the course of developing novel therapeutic agents for AD, anti-diabetic drugs were regarded as potential candidates.

Some studies has been reported the protective effects of metformin (Met) [7–9], the commonly used oral hypoglycemic, and saxagliptin (Saxa) [10], the member of gliptins class that is considered as one of the newest classes of oral hypoglycemic, against streptozotocin (STZ)-induced dementia in rats. On the other hand, it has been shown recently that Met increases beta amyloid (Aβ) expression in neuronal cultures [11]. However, the effects of Met and Sax on the actual markers of AD, like the levels of phosphorylated tau, the accumulation of amyloid plaques, neurotransmitter levels as well as learning and memory functions in insulin resistant senile rats, have not been reported before.

The present study aimed to verify type 3 diabetes hypotheses in senile rats using the D-gal model of experimental aging, rather than the commonly used STZ model, as a mean to induce memory and learning deficits. The aim was extended to evaluate the efficacy of Met and Saxa in reducing D-gal-induced AD and brain aging.

## Materials and methods

### Animals

Adult male albino Wistar rats, weighing 200–250g, were obtained from the animal house colony of National Research Centre (NRC, Egypt). The animals were maintained at a controlled temperature of 24 ± 1°C with a 12–12 h light-dark cycle (light cycle, 07:00–19:00). They were allowed free access to water and standard chow *ad libitum*. This study has been approved by the ethics committees of the NRC and Faculty of Pharmacy, Cairo University (Egypt). All procedures and experiments were performed according to the protocol approved by them, and the animals were treated according to the national and international ethics guidelines. The earliest scientifically justified endpoint was used in this study to prevent pain or distress in the experimental animals.

### Drugs and chemicals

Synthetic D-gal and Saxa powder were purchased from Sigma-Aldrich (USA) and Astrazeneca Pharmaceuticals (Egypt), respectively. Met was graciously gifted to us by Merck Pharmaceuticals (Egypt).

### Experimental design

Forty-eight rats were randomly allocated into six groups (n = 8). For 60 successive days, the rats of the first three groups received plain distilled water (1.5 ml/kg/day, s.c) and served as a control set, while the other three groups received D-gal dissolved in distilled water (150 mg/kg/day, s.c [12] and served as D-gal-treated set. On day 61, the animals started to receive the treatments for 30 successive days simultaneously with the continuation of the D-gal injections until the end of the experiment, which lasted for 90 days. Treatment of the animals was as follows: In both sets, the 1^st^ group received distilled water orally, while the other 2 groups received Met (500 mg/kg/day, p.o.) [13] or Saxa (1 mg/kg/day, p.o) [14], respectively.

### Assessment of the rats' body weight

The weight of each rat was measured every week throughout the whole experiment. Results were presented as a percent of the mean weight of the normal group.

### Assessment of spontaneous locomotor activity of rats

Spontaneous locomotor exploratory behavior of the animals was measured by detecting the rats`movements using a grid floor activity cage (Model no. 7430, Ugo-Basile, Comerio, Italy).

The experiment was carried out on the last day of injection and the rats were tested only once. In the test day rats were removed from their home cages and placed in the activity-monitoring chamber which is a Plexiglas chamber that measures 44 (width)×44 (length)×20 (height) cm, includes 10 pairs of infrared photocells, which were used to measure the horizontal locomotor activity. Consecutive interruption of two beams was recorded as one unit of locomotor activity. Interruption information was processed by the activity cage software to provide counts of horizontal movements. Animals were placed for 10 min in the monitoring chamber to detect changes in spontaneous locomotor activity [15].

### Measurement of spatial memory and learning by Morris water maze (MWM)

Twenty-four hours following the last day of injections (the 91^st^ day), the rats were acclimatized for 1 h in the test room before placing the animals in the MWM. The whole experiment lasted for five successive days [16]. The room was arranged such that the animal being tested cannot see the experimenter during testing. On the first day of the experiment, the platform was kept visible by leaving the water transparent and placing a flag on the platform to increase its visibility; platform position and the position of the rat dropping in the pool were changed for each trial. Each animal was trained five times to reach the platform with an inter-trial interval of no more than 20 min. On the second, third, and fourth days, the water was rendered opaque by adding powdered skimmed milk to the pool and the platform was painted white to be hidden. The testing procedure was repeated with the platform at a fixed certain quadrant of the pool about four cm below water for all trails. The time required for the animal to reach the platform was recorded.

On the fifth (final) day of the experiment, only one testing episode was required for each animal. The platform was removed from the pool, the animal was placed in the pool from one fixed dropping position, and the time spent with each animal in the target quadrant (the quadrant that used to retain the platform) was then calculated.

### Estimation of blood glycated hemoglobin (HbA1c) level

Blood samples were withdrawn from rats of all groups via the retro-orbital plexus vein under light ether anesthesia 24 h after the end of MWM [17]. The blood HbA1c level was estimated using a commercial reagent kit (Biodiagnostic, Egypt).

### Brain tissues biochemical analysis

Immediately after blood sampling, animals were sacrificed by cervical dislocation under ether anesthesia. The brain from each rat was immediately dissected out, and rinsed with phosphate-buffered saline (PBS) to remove excess blood. Weighed brains were homogenized (MPW-120 homogenizer, med instruments, Poland) in PBS to obtain 20% homogenate. The homogenates were centrifuged for 5 min at 5000 x g using a cooling centrifuge (Sigma and laborzentrifugen, 2k15, Germany). The supernatant was taken immediately and stored overnight at ≤ –80°C.

The supernatant was then assayed for the contents of lipid peroxides measured as malondialdehyde (MDA), reduced glutathione (GSH), and NOx (nitrite and nitrate, stable metabolites of NO), using commercial reagent kits (Biodiagnostic, Egypt) [18–20]. Moreover, whole brain content of tumor necrosis factor alpha (TNF-α) was assessed using specific diagnostic kit (RayBiotech, USA) [21].

Markers of insulin resistance such as insulin level, insulin receptors, receptor for advanced glycation end products (RAGE), as well as acetylcholine and glutamate were also assessed using specific diagnostic kits (RayBiotech, USA), (cusabio biotech, USA), (Sigma-Aldrich Company, USA), and (Abnova, United Kingdom) [22, 23].

The level of phosphorelated tau as a marker of AD severity was also assayed according to the method described by the manufacturer`s protocol (RayBiotech, USA).

### Histopathological examination

Two brains from each group were fixed in 10% neutral buffered formalin for at least 72 h, washed, dehydrated, and embedded in paraffin. Afterwards, brains were sectioned coronally at sections of 4μm thick. Sections were stained with hematoxylin and eosin (H&E) for routine histopathological examination, and Congo red for the demonstration of amyloid plaques. Five sections per group were examined using a binocular Olympus CX31 microscope (UK).

For assessment of neuronal loss, the degenerated and/or necrotic neurons in the CA1, CA2, and CA3 subdivisions of the hippocampus were counted in five high microscopic field (X40) per group according to the method of West *et al* [24] with some modifications. Amyloid plaques were counted in ten random low microscopic field (X10) in Congo red-stained sections according to the method of Snowdon [25] with some modifications. The obtained data were then statistically analyzed.

### Immunohistochemical analysis

For demonstration of reactive astrocytes surrounding amyloid plaques, immunohistochemical procedures were used to recognize glial fibrillary acidic protein (GFAP) immune reactive cells in paraffin sections according to the method of Hol *et al* [26]. After deparaffinization in toluene, the tissue sections were rehydrated in ethanol, and then incubated with H_2_O_2_ for blocking the endogenous peroxidase activity. Anti-GFAP antibody (Dako) was used as botinylated primary antibody. The immune reactive cells were visualized using the chromogen diaminobenzidine tetrahydrochloride (DAB) (Sigma, USA).

### Statistical analysis

All the values are presented as means ± standard error of the means (SE). Comparisons between different groups were carried out using one-way analysis of variance (ANOVA) followed by Tukey test for multiple comparisons except for the learning curve of Morris water maze where two way ANOVA was used [27]. Graphpad Prism software, version 5 (USA) was used to carry out these statistical tests. The difference was considered significant when *p* ˂ 0.05.

## Results

### The body weight of the rats

**Fig 1:**
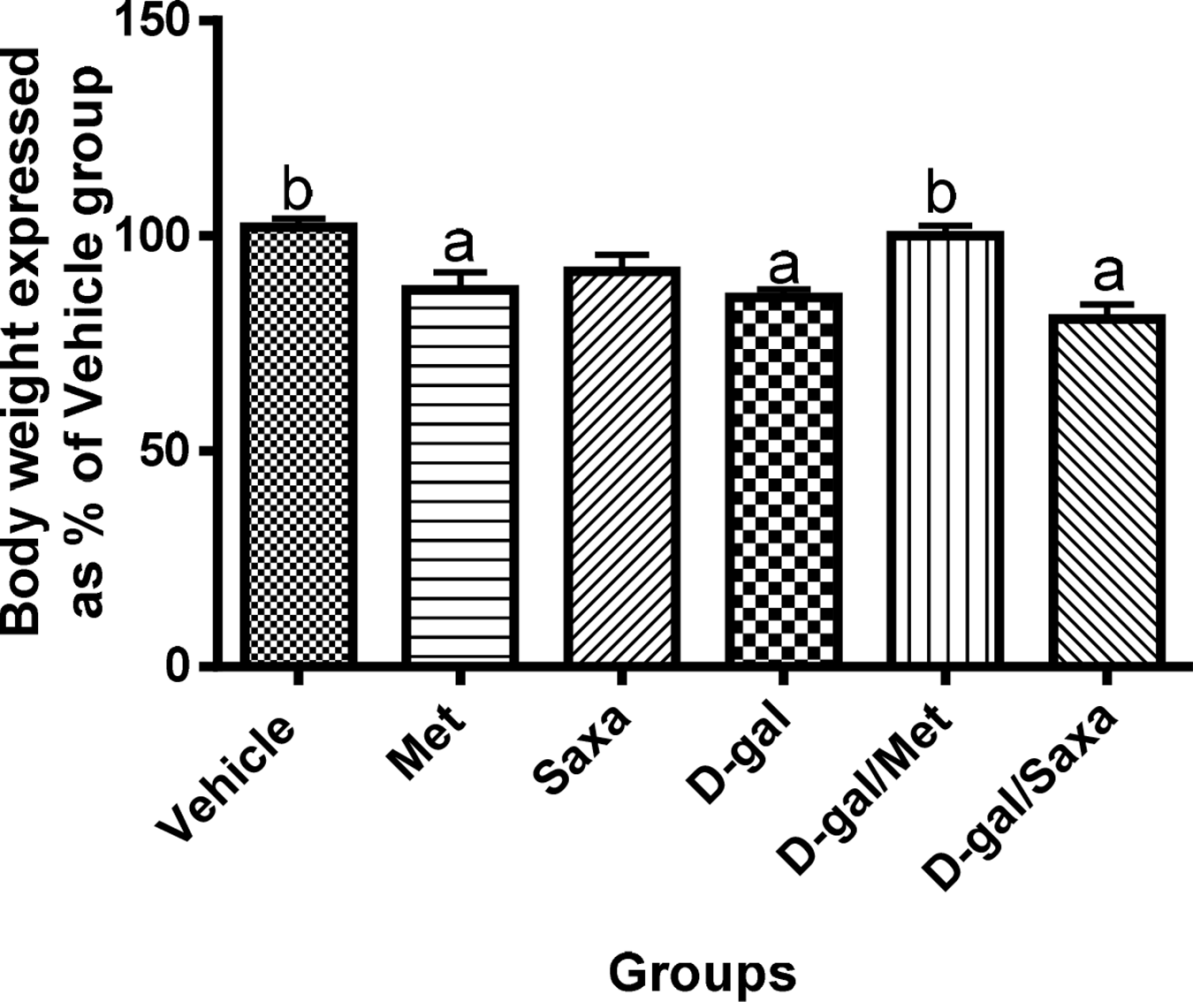
The body weight of the rats. Vehicle, rats treated with distilled water (1.5 ml/kg/day, s.c); Met, rats treated with metformin (500 mg/kg/day, p.o); Saxa, rats treated with saxagliptin (1 mg/kg/day, p.o); D-gal, rats treated with D-galactose (150 mg/kg/day, s.c); D-gal/Met, rats treated with D-galactose and metformin; D-gal/Saxa, rats treated with D-galactose and saxagliptin. Each value represents the mean weight as a percent of the mean weight of the Vehicle group ± S.E. for each group; n = 8. ^a^ Significantly different from Vehicle group at *p* < 0.05. ^b^ Significantly different from D-gal group at *p* < 0.05.

Administration of D-gal (150 mg/kg/day, s.c) for 90 days significantly reduced the weight of the rats as compared to the normal group. Met administration also resulted in a significant decrease in rats`weight, while Saxa showed no effect on rats`weight. On the other hand, treatment of the animals with Met following D-gal injections regained the normal weight of the animals; however Sax failed **(Fig 1)**.

### Spontaneous locomotor activity of rats

**Fig 2:**
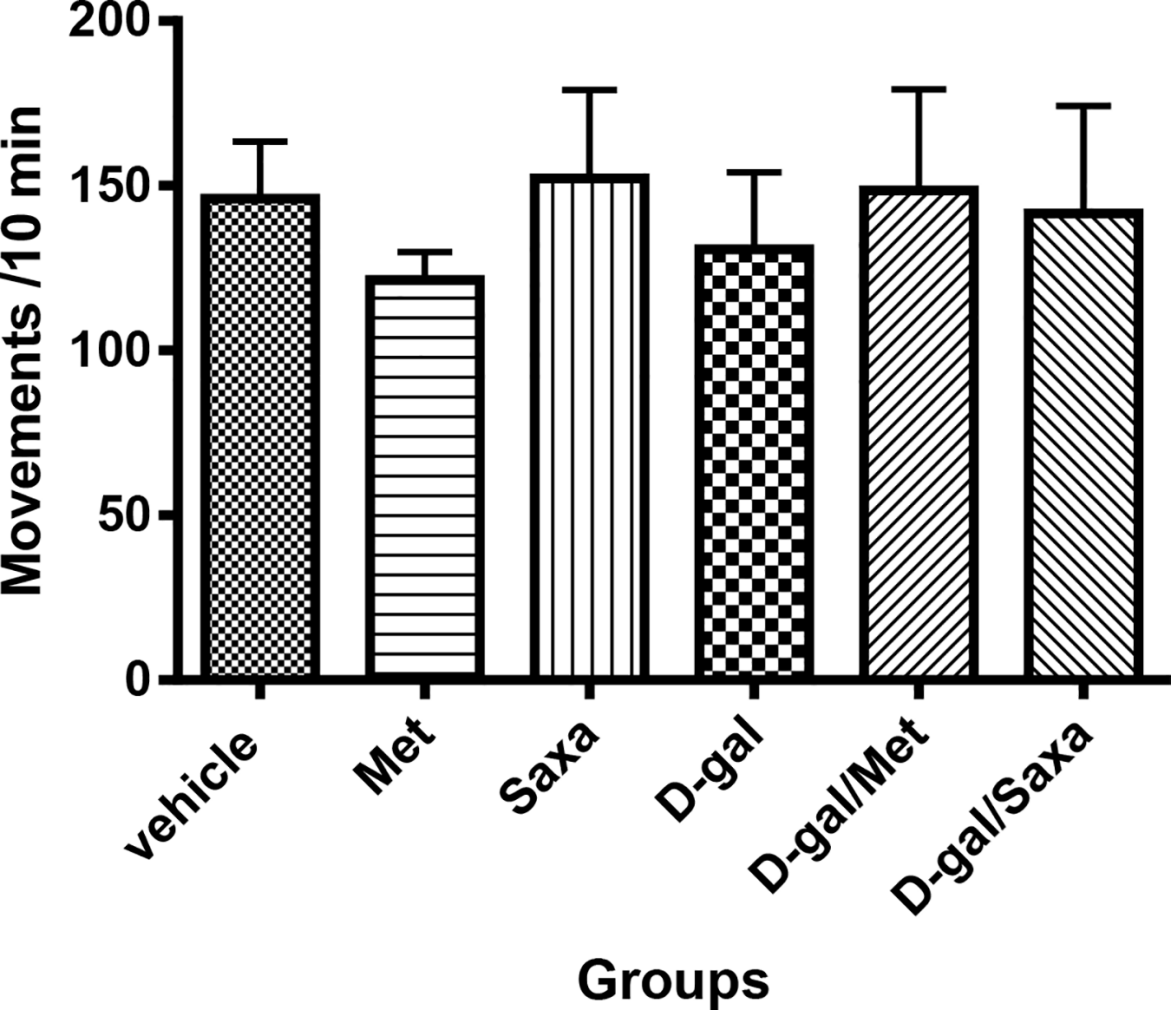
Spontaneous locomotor activity of rats. Vehicle, rats treated with distilled water (1.5 ml/kg/day, s.c); Met, rats treated with metformin (500 mg/kg/day, p.o); Saxa, rats treated with saxagliptin (1 mg/kg/day, p.o); D-gal, rats treated with D-galactose (150 mg/kg/day, s.c); D-gal/Met, rats treated with D-galactose and metformin; D-gal/Saxa, rats treated with D-galactose and saxagliptin. Each value represents the mean counts per 10 min as an indication of the rats spontaneous locomotor activity ± S.E. for each group (n = 8).

Data revealed no significant differences between groups. This was regarded as further proof that the animals did not develop any visual or locomotor changes in response to treatments that could affect the results of Morris water maze later on, ensuring that any observed differences in Morris water maze results could only be attributed to memory and learning impairments **(Fig 2)**.

### The mean escape latency time in MWM

On the first day of training, there were no significant differences between groups in the mean escape latency.

On the second, third and fourth days of investigation, treatment of animals with Met and Saxa alone did not induce any changes, however, D-gal significantly increased the mean escape latency time of the rats as compared to the normal control group.

Treatment of the D-gal-injected animals with Met resulted in a significant decrease in the mean escape latency time as compared to D-gal group. Though, Saxa showed no significant improvement of the D-gal-induced effect on the escape latency time on the second day, but significantly decreased it to normal levels on the last two days **(Table 1**).

**Table 1:** The mean escape latency time in Morris water maze.

Groups	Mean escape latency time in MWM (sec)			
	1^st^ day	2^nd^ day	3^rd^ day	4^th^ day
**Vehicle**	45.57±1.26	31.11^b^±1.40	22.125 ^b^±2.32	16.84 ^b^±2.05
**Met**	45.16±2.14	32.47^b^±2.70	25.27^b^±2.07	18.83^b^±2.06
**Saxa**	47.11± 1.61	27.37^b^±1.92	21.27^b^±2.69	16.90^b^±3.02
**D-gal**	51.35±1.53	45.36^a^±1.16	39.51^a^±1.79	33.80^a^±1.62
**D-gal/Met**	46.91±1.28	34.11^b^±2.08	21.23^b^±1.47	14.42^b^±1.18
**D-gal/Saxa**	50.57± 1.99	41.54^a^±2.24	29.75^b^±2.41	22.88^b^±2.49

MWM, Morris water maze; Vehicle, rats treated with distilled water (1.5 ml/kg/day, s.c); Met, rats treated with metformin (500 mg/kg/day, p.o); Saxa, rats treated with saxagliptin (1 mg/kg/day, p.o); D-gal, rats treated with D-galactose (150 mg/kg/day, s.c); D-gal/Met, rats treated with D-galactose and metformin; D-gal/Saxa, rats treated with D-galactose and saxagliptin. Each value represents mean ± S.E; n = 8.

^a^ Significantly different from Vehicle group on the corresponding day at *p* < 0.05.

^b^ Significantly different from D-gal group on the corresponding day at *p* < 0.05.

### The mean time spent in the target quadrant in MWM

D-gal significantly decreased the mean time spent by the rats in the target quadrant to reach a value of 18.63 sec as compared to 34 sec in the normal control group, indicating impaired learning and memory function.

**Fig 3:**
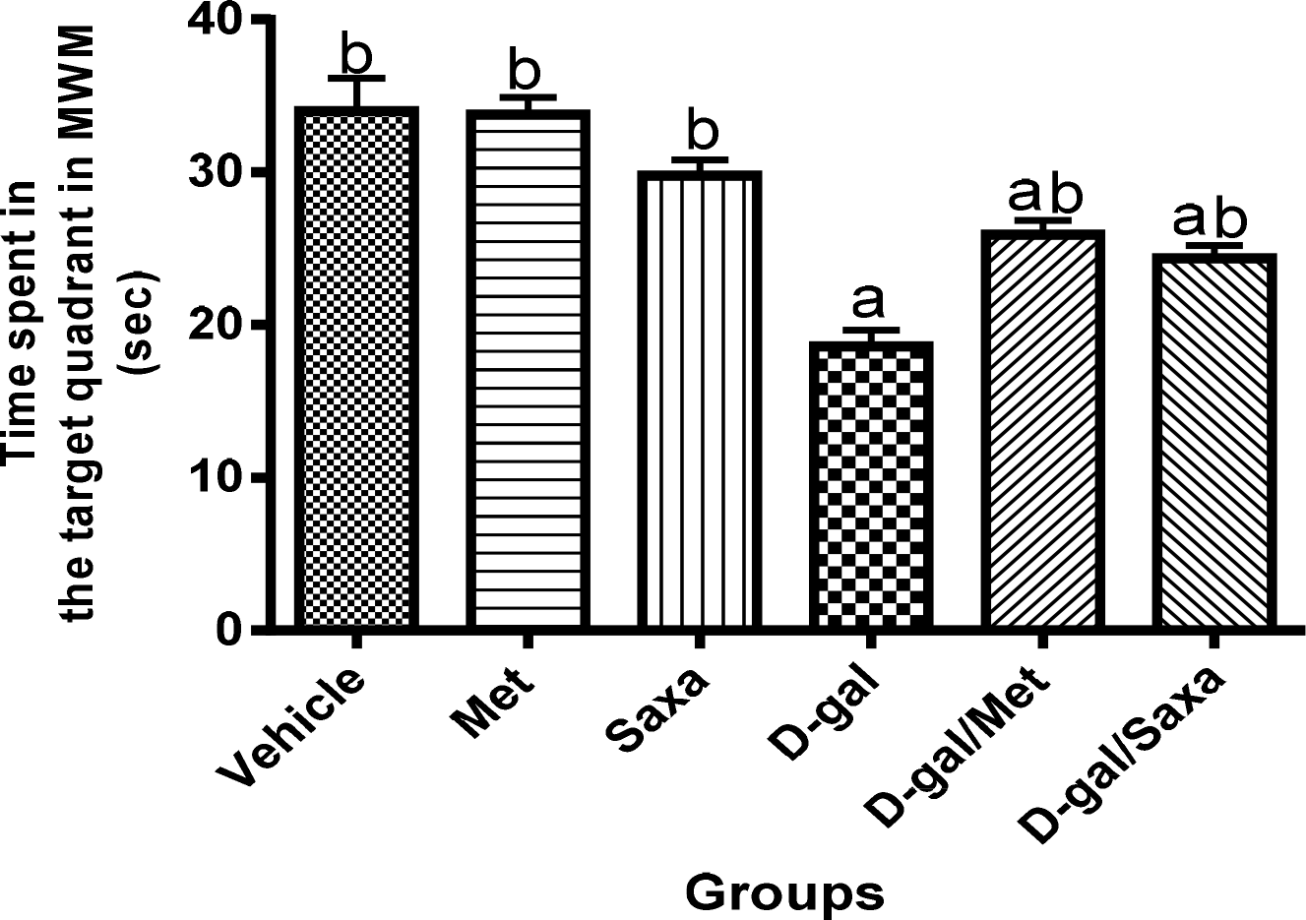
The mean time spent in the target quadrant in Morris water maze. MWM, Morris water maze; Vehicle, rats treated with distilled water (1.5 ml/kg/day, s.c); Met, rats treated with metformin (500 mg/kg/day, p.o); Saxa, rats treated with saxagliptin (1 mg/kg/day, p.o); D-gal, rats treated with D-galactose (150 mg/kg/day, s.c); D-gal/Met, rats treated with D-galactose and metformin; D-gal/Saxa, rats treated with D-galactose and saxagliptin. Each value represents mean ± S.E; n = 8. ^a^ Significantly different from Vehicle group at *p* < 0.05. ^b^ Significantly different from D-gal group at *p* < 0.05.

The mean time spent by rats treated with Met and Saxa in the target quadrant was found to be normal. However, treatment of the animals with either Met or Saxa following D-gal injection resulted in a significant increase in mean time spent in the target quadrant as compared to D-gal control group to reach values of 25.91 and 24.38 sec, respectively, indicating an improvement of learning and memory function **(Fig 3)**.

### The blood HbA1c level

A significant increase in the blood level of HbA1c was observed in rats treated with D-gal (32.64 ng/l compared to 8.10 ng/l in the normal group). Met and Sax showed no effect on the normal blood level of HbA1c.

**Fig 4:**
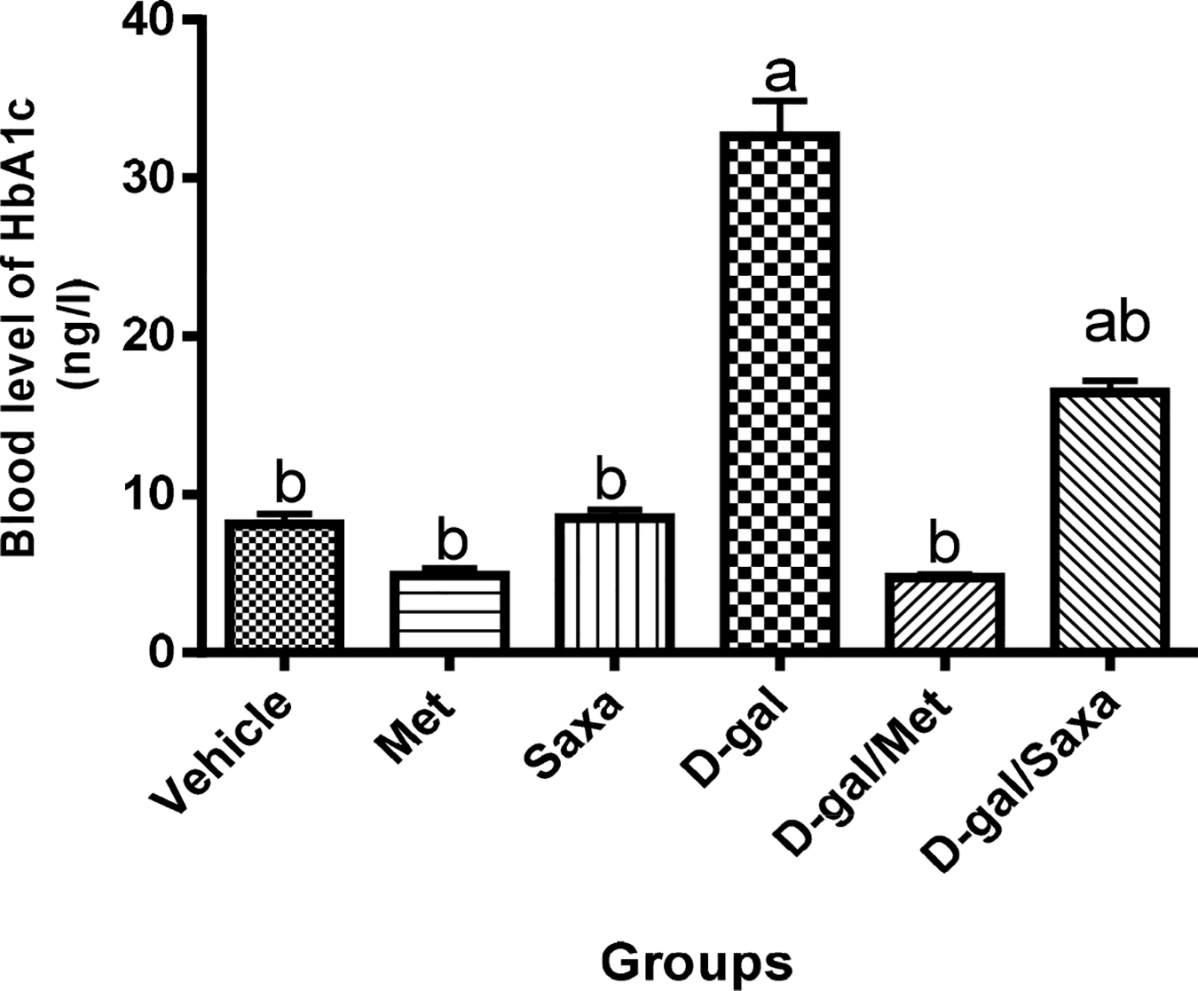
Blood glycated hemoglobin level. HbA1c, glycated hemoglobin; Vehicle, rats treated with distilled water (1.5 ml/kg/day, s.c); Met, rats treated with metformin (500 mg/kg/day, p.o); Saxa, rats treated with saxagliptin (1 mg/kg/day, p.o); D-gal, rats treated with D-galactose (150 mg/kg/day, s.c); D-gal/Met, rats treated with D-galactose and metformin; D-gal/Saxa, rats treated with D-galactose and saxagliptin. Each value represents mean ± S.E; n = 8. ^a^ Significantly different from Vehicle group at *p* < 0.05. ^b^ Significantly different from D-gal group at *p* < 0.05.

However, Met normalized the blood level of HbA1c in the D-gal-treated rats, while, Saxa resulted in a significant decrease in that level **(Fig 4)**.

### The brain oxidative stress biomarkers

Treatment of rats with either Met or Saxa did not affect the normal brain contents of MDA and GSH. On the other hand, the brain content of MDA was significantly increased in D-gal-treated rats to 4.24-fold that of the normal group, and a significant decrease in GSH content was detected.

**Fig 5:**
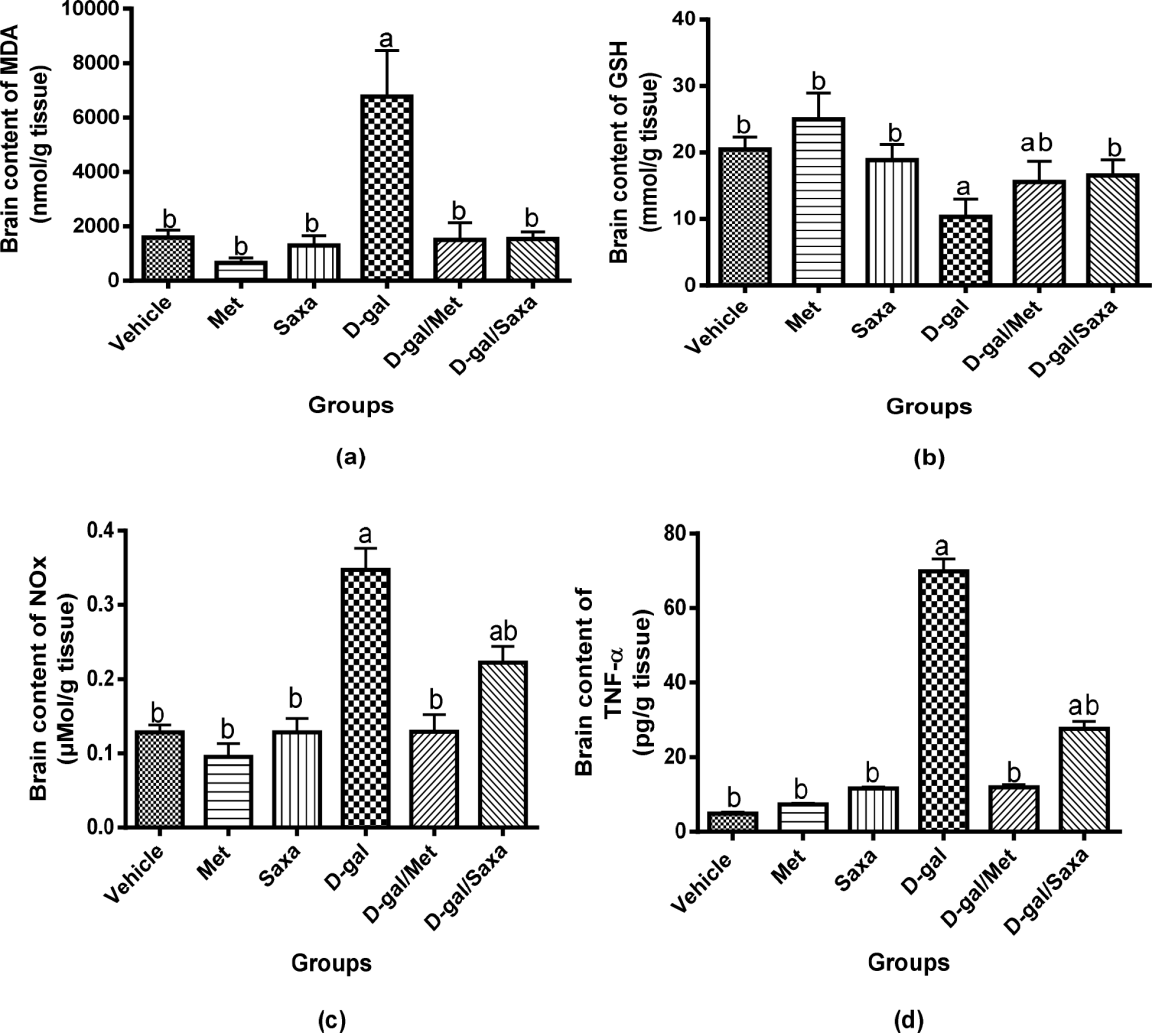
Brain contents of MDA, GSH, NOx, and TNF-α. MDA, malondialdehyde; GSH, reduced glutathione; NOx, nitrite and nitrate, stable metabolites of NO; TNF-α, tumor necrosis factor alpha; Vehicle, rats treated with distilled water (1.5 ml/kg/day, s.c); Met, rats treated with metformin (500 mg/kg/day, p.o); Saxa, rats treated with saxagliptin (1 mg/kg/day, p.o); D-gal, rats treated with D-galactose (150 mg/kg/day, s.c); D-gal/Met, rats treated with D-galactose and metformin; D-gal/Saxa, rats treated with D-galactose and saxagliptin. Each value represents mean ± S.E; n = 6. ^a^ Significantly different from Vehicle group at *p* < 0.05. ^b^ Significantly different from D-gal group at *p* < 0.05.

Treatment of rats with either Met or Saxa significantly retrieved D-gal induced altered level of MDA. Met also corrected the brain content of GSH in those rats and Saxa resulted in a marked improvement **(Fig 5A and 5B)**.

### The brain contents of NOx and TNF-α

D-gal significantly increased NOx and TNF-α content in brain homogenate of the rats to be 0.35 μmol/g and 69.92 pg/g, respectively, as compared to 0.128 μmol/g and 4.89 pg/g, respectively, in the normal rats.

Met and Saxa showed no effect on the normal values of NOx and TNF-α in the normal rat. Moreover, normal brain contents of NOx and TNF-α was observed in rats treated with D-gal/Met. On the other hand, in contrast to D-gal group, Saxa markedly decreased the brain contents of NOx and TNF-α to 0.22 μmol/g and 27.64 pg/g, respectively, in D-gal-treated animals **(Fig 5C and 5D)**.

### Insulin resistance markers in the brain

Met and Saxa showed no effect on the normal values of insulin, insulin receptors, and RAGE in the normal rat. D-gal significantly increased insulin level in the rat brain homogenate to be 112.70 μIU/g as compared to 10.75 μIU/g in normal rats. Moreover, it increased RAGE brain level to 55.41 ng/g as compared to 3.50 ng/g in normal rats. On the other hand, D-gal significantly decreased insulin receptor levels to reach a value of 1.29 IU/g as compared to 18.47 IU/g in the normal group.

**Fig 6:**
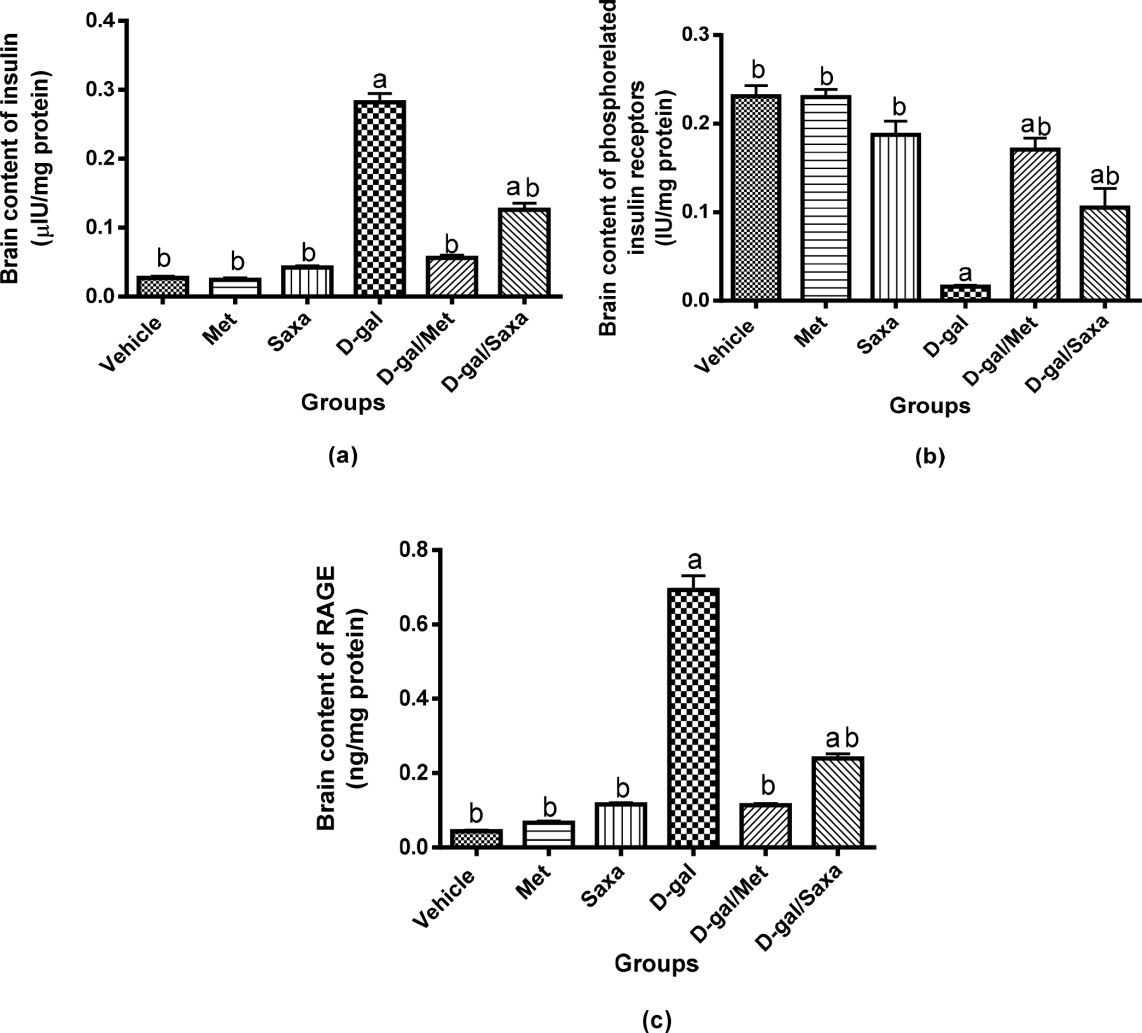
Insulin resistance markers in the brain. Vehicle, rats treated with distilled water (1.5 ml/kg/day, s.c); Met, rats treated with metformin (500 mg/kg/day, p.o); Saxa, rats treated with saxagliptin (1 mg/kg/day, p.o); D-gal, rats treated with D-galactose (150 mg/kg/day, s.c); D-gal/Met, rats treated with D-galactose and metformin; D-gal/Saxa, rats treated with D-galactose and saxagliptin. Each value represents mean ± S.E; n = 6. ^a^ Significantly different from Vehicle group at *p* < 0.05. ^b^ Significantly different from D-gal group at *p* < 0.05.

Administration of Met or Saxa to D-gal-treated rats significantly decreased insulin and RAGE levels in the brain homogenate as compared to D-gal group, while the treatment resulted in an elevation of insulin receptors level. (**Fig 6A, 6B and 6C**).

### The brain contents of acetylcholine and glutamate

A significant decrease in acetylcholine and glutamate contents in the brain was observed in rats treated with D-gal; their concentrations were 2.62 nmol/g and 0.62 mmol/g, respectively, compared to normal values of 34.07 nmol/g and 4.39 mmol/g, respectively.

**Fig 7:**
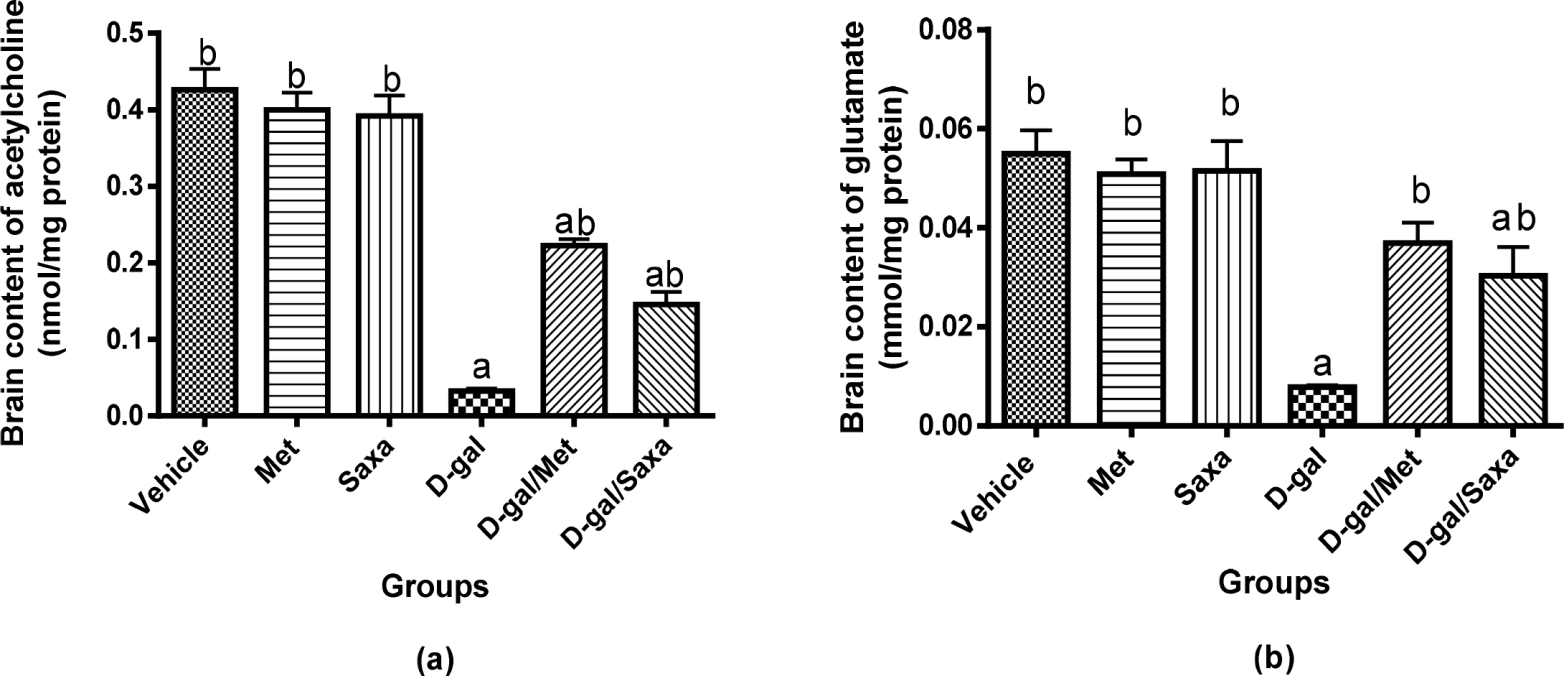
Brain contents of neurotransmitters, acetylcholine and glutamate. Vehicle, rats treated with distilled water (1.5 ml/kg/day, s.c); Met, rats treated with metformin (500 mg/kg/day, p.o); Saxa, rats treated with saxagliptin (1 mg/kg/day, p.o); D-gal, rats treated with D-galactose (150 mg/kg/day, s.c); D-gal/Met, rats treated with D-galactose and metformin; D-gal/Saxa, rats treated with D-galactose and saxagliptin. Each value represents mean ± S.E; n = 6. ^a^ Significantly different from Vehicle group at *p* < 0.05. ^b^ Significantly different from D-gal group at *p* < 0.05.

Met and Saxa showed no effect on the normal values of both neurotransmitters in the normal rat. However, they significantly increased acetylcholine and glutamate contents in brain homogenate of rats injected with D-gal in contrast with D-gal-treated rats. Interestingly, a normal brain content of glutamate was observed in D-gal/Met group **(Fig 7A and 7B).**

### The brain content of phosphorelated tau

Met and Saxa showed no effect on the normal brain content of phosphorelated tau in the normal rats. D-gal significantly increased the brain content of phosphorelated tau the rats to 73.44 pg/g compared to 10.93 pg/g in the normal ones.

**Fig 8:**
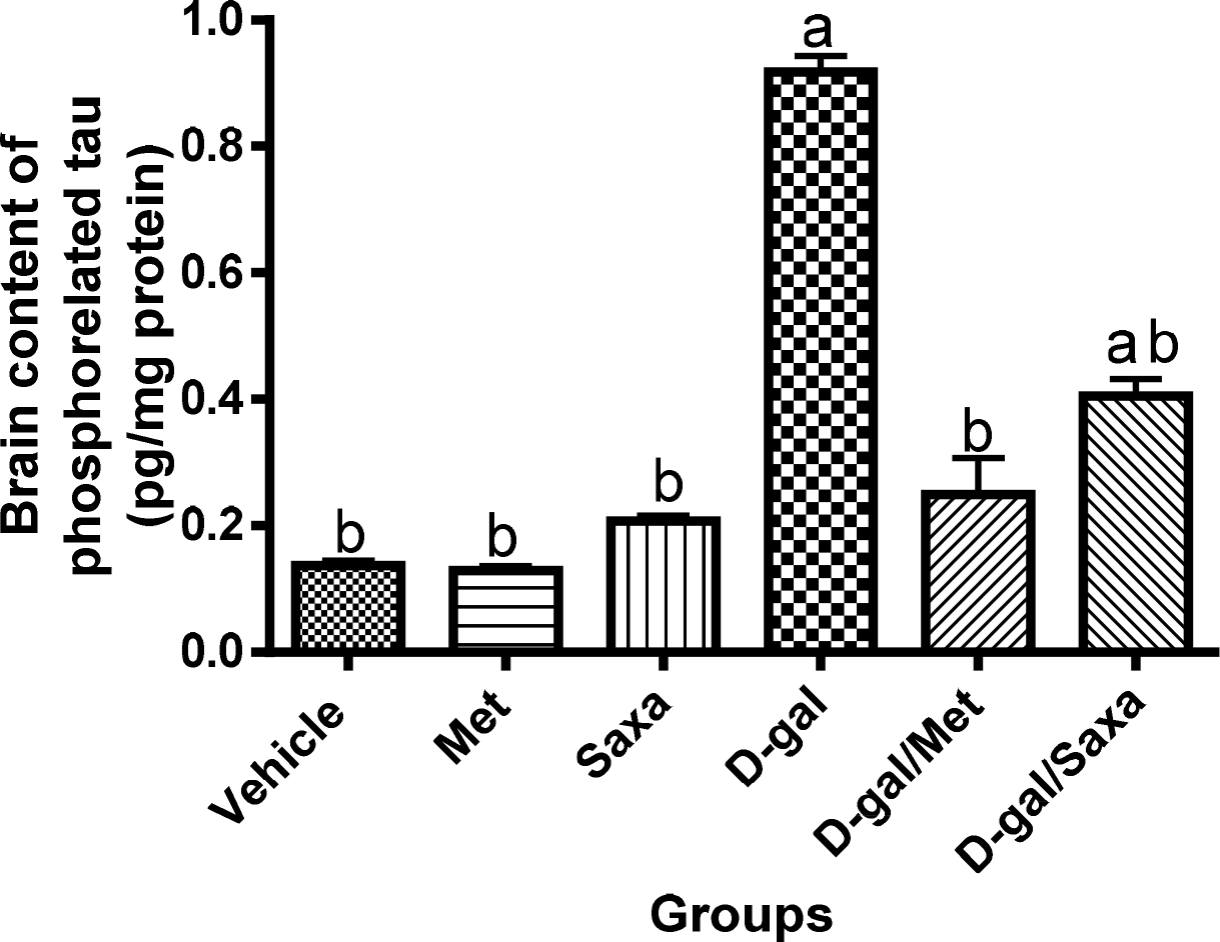
Phosphorelated tau concentration in the brain homogenate. Vehicle, rats treated with distilled water (1.5 ml/kg/day, s.c); Met, rats treated with metformin (500 mg/kg/day, p.o); Saxa, rats treated with saxagliptin (1 mg/kg/day, p.o); D-gal, rats treated with D-galactose (150 mg/kg/day, s.c); D-gal/Met, rats treated with D-galactose and metformin; D-gal/Saxa, rats treated with D-galactose and saxagliptin. Each value represents mean ± S.E; n = 6. ^a^ Significantly different from Vehicle group at *p* < 0.05. ^b^ Significantly different from D-gal group at *p* < 0.05.

Administration of either Met or Saxa to D-gal-treated animals significantly decreased the unregulated brain content phosphorelated tau to reach values of 19.97 and 32.42 pg/g, respectively (**Fig 8**).

### Histopathological changes in brain tissues

**Fig 9:**
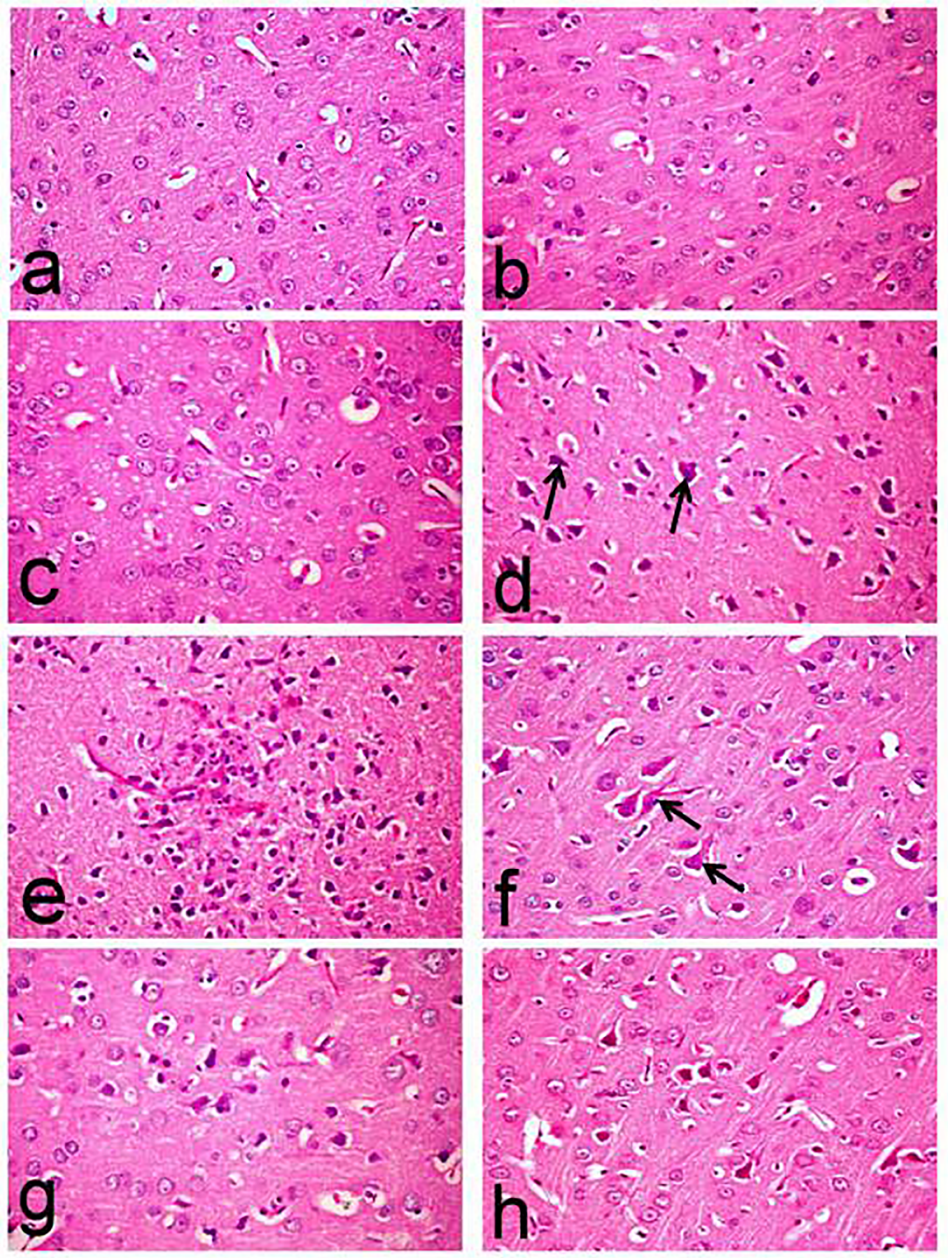
Histopathological changes in the cerebral cortex of rats. The micrographs represent the following groups: **(a)** Vehicle, **(b)** Met, **(c)** Saxa, **(d, e, f)** D-gal, **(g)** D-gal/Met, and **(h)** D-gal/Saxa. (H&E, X40). Vehicle, rats treated with distilled water (1.5 ml/kg/day, s.c); Met, rats treated with metformin (500 mg/kg/day, p.o); Saxa, rats treated with saxagliptin (1 mg/kg/day, p.o); D-gal, rats treated with D-galactose (150 mg/kg/day, s.c); D-gal/Met, rats treated with D-galactose and metformin; D-gal/Saxa, rats treated with D-galactose and saxagliptin. **(a,b,c)** showing normal histological structure; **(d,e,f)** showing neuronal degeneration associated with neuronophagia in which the degenerated neurons appeared shrunken and surrounded by microglia cells (arrow) **(d)**, amyloid plaque surrounded by astrocytes and microglia cells **(e)** and flame shape neurofibrillary tangles (arrow) **(f)**; **(g)** showing decreased number of degenerated neurons; **(h)** showing neuronal degeneration.

**Fig 10:**
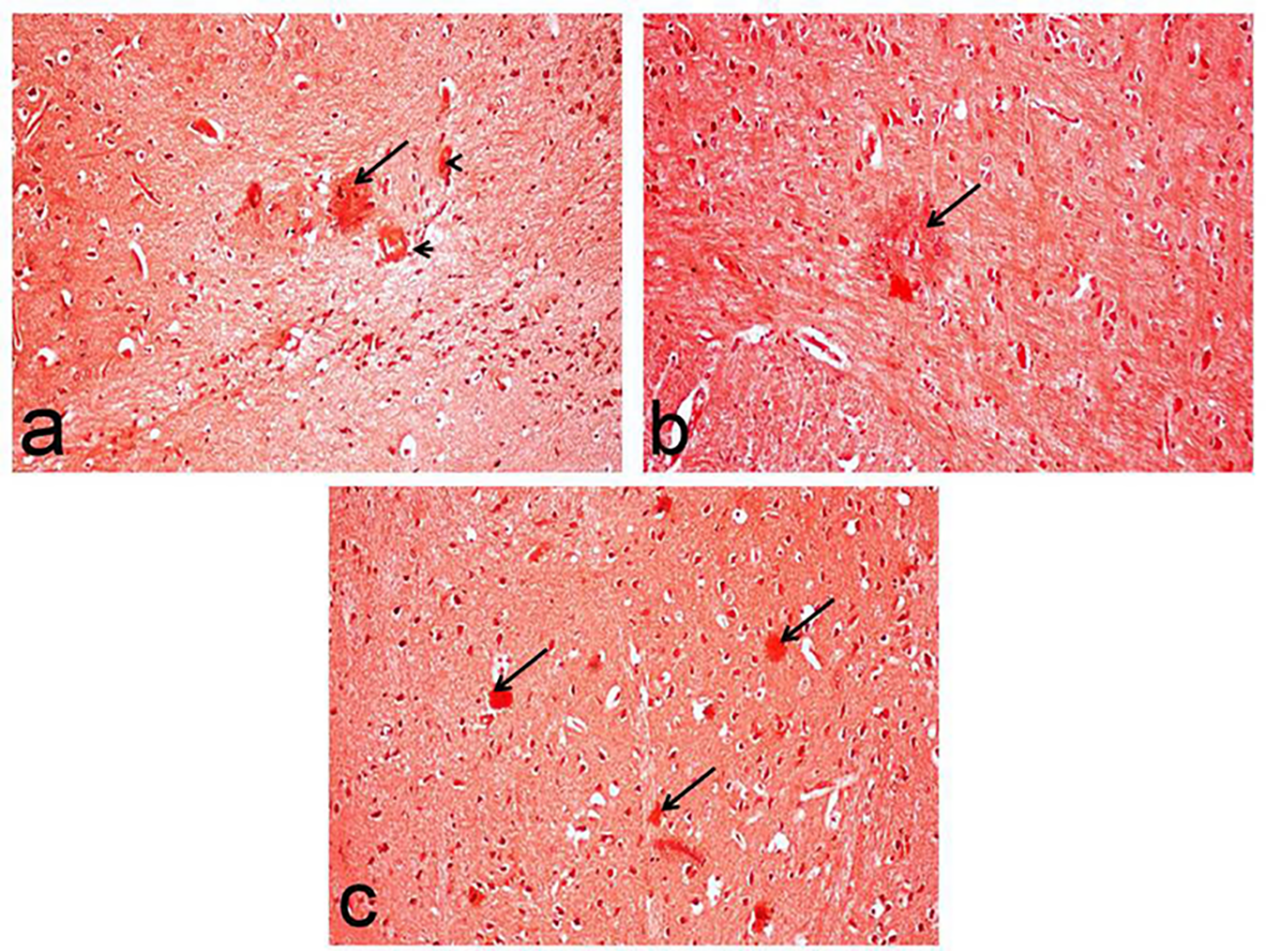
Congo red-stained brain sections of rats for visualization of amyloid plaques. The micrographs represent the following groups: **(a)** D-gal-treated group showing red amyloid plaques (arrow) associated with cerebral amyloid angiopathy (arrow head), **(b)** D-gal/Met group showing sparse deposition of amyloid plaque (arrow), and **(c)** D-gal/Saxa group showing multiple amyloid plaques (arrows). (Congo red stain, X20). D-gal, rats treated with D-galactose (150 mg/kg/day, s.c); D-gal/Met, rats treated with D-galactose and metformin; D-gal/Saxa, rats treated with D-galactose and saxagliptin.

Table 2 illustrates the number of degenerated and or necrotic neurons and amyloid plaques recorded in the brain of control and treated rats. The brain tissue of normal rats revealed normal histological structure with normal cerebral cortex and hippocampus region (**Figs 9A and 10A**). Similarly, the brain of Met and Saxa groups revealed normal morphology of the cerebral cortex (**Fig 9B and 9C)** and hippocampus region (**Fig 10B and 10C**) with no histological abnormalities. No amyloid plaque deposition was demonstrated in the brain tissue of control, Met and Saxa groups. Whereas, the brain of D-gal-treated group showed deleterious histopathological alterations particularly in the cerebral cortex, which revealed wide spread neuronal degeneration associated with neuronophagia in which the degenerated neurons appeared shrunken and surrounded by microglia cells (**Fig 9D**).

**Table 2:** The number of degenerated and or necrotic neurons and amyloid plaques recorded in the brain of the rats.

Groups	Number of degenerated and/or necrotic neurons (high microscopic field)	Number of amyloid plaques (low microscopic field)
**Vehicle**	0.40^b^±0.24	—-
**Met**	0.20^b^±0.20	—-
**Saxa**	0.40^b^ ±0.20	—-
**D-gal**	17.40^a^±2.50	41.60^a^±0.87
**D-gal/Met**	8.00^ab^±1.14	14.00^ab^±0.32
**D-gal/Saxa**	11.80^ab^±1.07	24.40^b^±0.24

Vehicle, rats treated with distilled water (1.5 ml/kg/day, s.c); Met, rats treated with metformin (500 mg/kg/day, p.o); Saxa, rats treated with saxagliptin (1 mg/kg/day, p.o); D-gal, rats treated with D-galactose (150 mg/kg/day, s.c); D-gal/Met, rats treated with D-galactose and metformin; D-gal/Saxa, rats treated with D-galactose and saxagliptin.

One of the most characteristic lesions demonstrated in this group was the deposition of amyloid plaque with eosinophilic core surrounded by astrocytes and microglia cells (**Fig 9E**). Marked increase of amyloid plaque deposition (41.60±0.87) was recorded in this group. These plaques, which appeared red in Congo red-stained sections, were widely distributed in the cerebral cortex and hippocampus regions. They were associated with cerebral amyloid angiopathy with deposition of amyloid β protein in the wall of meningeal and cerbrocortical blood vessels (**Fig 10A**).

**Fig 11:**
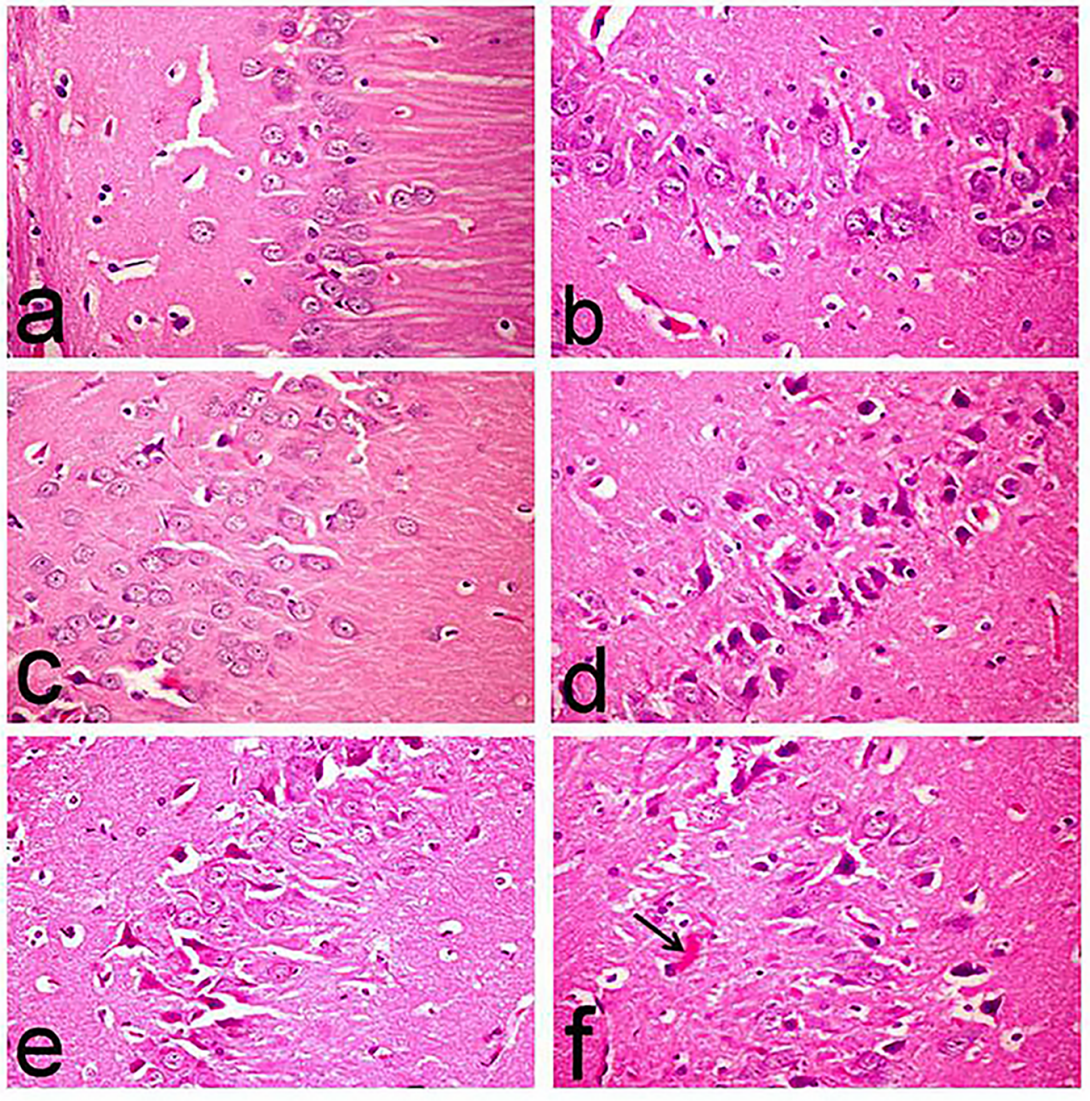
Histopathological changes in the hippocampi of rats. The micrographs represent the following groups: **(a,b,c)** Vehicle, Met, and Saxa, respectively, showing normal histological structure, **(d)** D-gal showing marked degeneration of neuronal cells associated with neuronophagia (arrow), **(e)** D-gal/Met group showing decreased number of degenerated neurons, and **(f)** D-gal/Saxa group showing degeneration of pyramidal neuronal cells associated with amyloid plaques deposition (arrow). (H&E, X40). Vehicle, rats treated with distilled water (1.5 ml/kg/day, s.c); Met, rats treated with metformin (500 mg/kg/day, p.o); Saxa, rats treated with saxagliptin (1 mg/kg/day, p.o); D-gal, rats treated with D-galactose (150 mg/kg/day, s.c); D-gal/Met, rats treated with D-galactose and metformin; D-gal/Saxa, rats treated with D-galactose and saxagliptin.

Flame shaped neurofibrillary tangles were another characteristic lesions demonstrated in this group (**Fig 9F**) in addition to astrogliosis. Moreover, hippocampus revealed marked degeneration of neuronal cells associated with neuronophagia (**Fig 11D**), and marked increase of degenerated and/or necrotic neurons (17.40±2.50), particularly in the CA1, CA2 and CA3 subdivisions, compared to the normal (0.40±0.24), Met (0.20±0.20), and Saxa (0.40±0.20) groups (**Fig 10A, 10B and 10C**, respectively).

On the contrary, these lesions were markedly regressed in the D-gal/Met group with decreased number of degenerated neurons in the cerebral cortex (**Fig 9G**), in addition to decreased number of deposited of amyloid plaque (14.00±0.32) (**Fig 10B**), compared to D-gal-treated group. Hippocampus of this group showed a decreased number of degenerated neurons (8.00±1.14) (**Fig 11E**) compared to the D-gal-treated one.

On the other hand, brain tissues of D-gal/Saxa group revealed mild improvement compared to D-gal/Met one, with presence of eosinophilic necrotic neurons (**Fig 9G**) and decreased number of amyloid plaques (24.40±0.24) (**Fig 10C**), which is significantly different from the D-gal-treated group. Reduced number of degenerated pyramidal neuronal cells (11.80±1.07) was also recorded in the hippocampus of this group (**Fig 11F**).

### Immunohistochemistry

**Fig 12:**
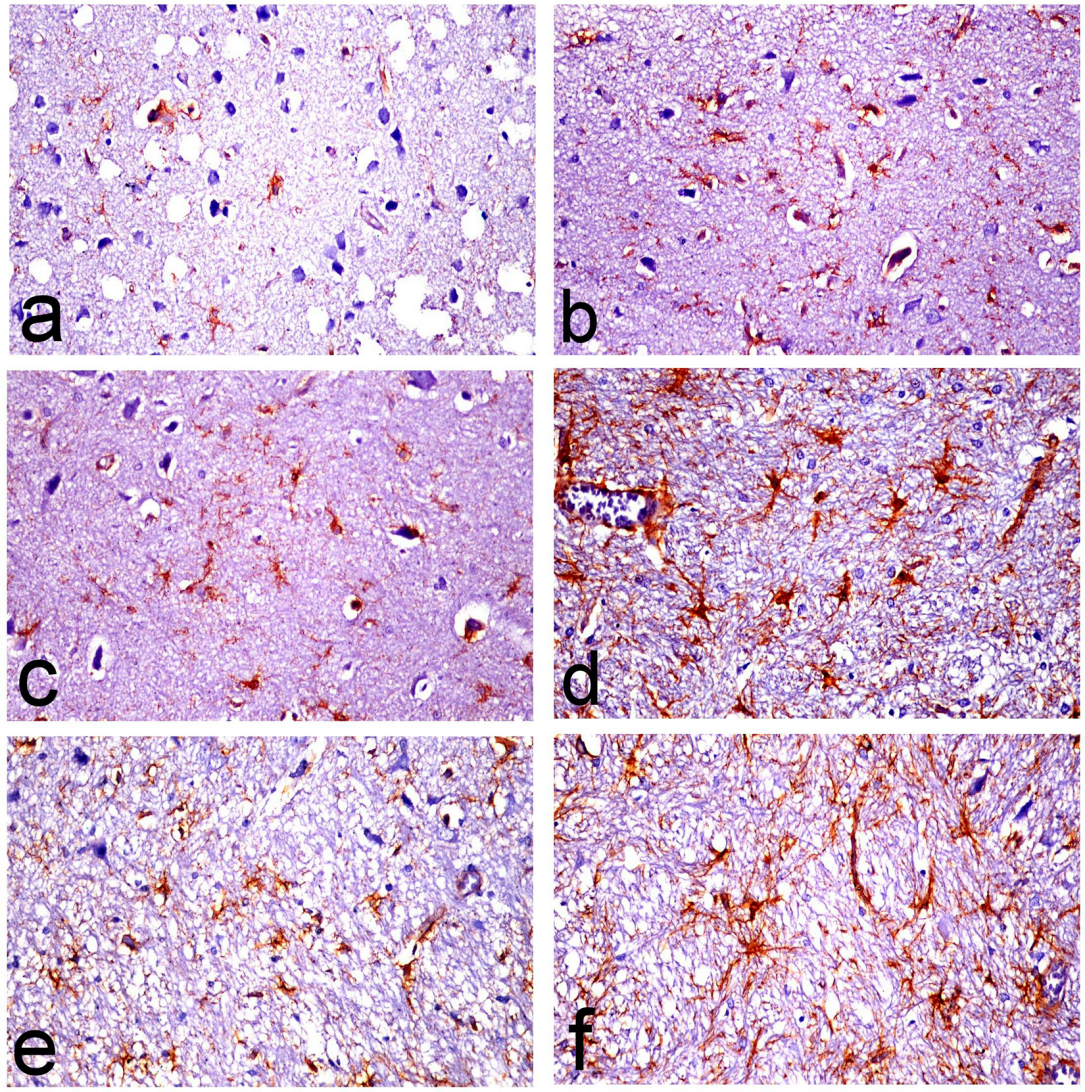
Glial fibrillary acidic protein (GFAP)-immune stained astrocytes in brain sections of rats. The micrographs represent the following groups: **(a)** Vehicle, **(b)** Met, **(c)** Saxa, **(d)** D-gal, **(e)** D-gal/Met, and **(f)** D-gal/Saxa. (GFAP immunohistochemical stain, X40). Vehicle, rats treated with distilled water (1.5 ml/kg/day, s.c); Met, rats treated with metformin (500 mg/kg/day, p.o); Saxa, rats treated with saxagliptin (1 mg/kg/day, p.o); D-gal, rats treated with D-galactose (150 mg/kg/day, s.c); D-gal/Met, rats treated with D-galactose and metformin; D-gal/Saxa, rats treated with D-galactose and saxagliptin.

Presence of reactive astrocytes surrounding amyloid plaques, appeared hypertrophied with increased thickness of their cytoskeletal processes was demonstrated in the D-gal-treated group (**Fig 12D**) compared to control, Met and Saxa groups (**Fig 12A, 12B and 12C**, respectively). The thickness of these processes was markedly decreased in the D-gal/Met group (**Fig 12E)** compared to D-gal-treated group. Mild improvement was recorded in D-gal/Saxa group (**Fig 12F)**.

## Discussion

The current study used D-gal to induce aging and senile-related AD in rats. Such model can produce aging in all organs of experimental animals, including the brain with symptoms that are particularly related to AD [28–30].

The present findings revealed that long-term administration of D-gal decreased the average weight of the animals; a result that could be attributed to aging and loss of muscle mass [29]. Met regained the normal weight of D-gal-injected animals. However, it decreased the average weight of the normal animals; this can be attributed to Met ability to reduce weight and counteract obesity [31].

In activity cage test, no significant change in the spontaneous locomotor activity of animals of all groups has been recorded. Conversely, findings of MWM test indicated that chronic administration of D-gal impaired the memory and learning capacity of rats. These findings are in accordance with previous findings in numerous literatures [32, 33]. Interestingly, the diversity between the findings of both performed behavioral tests could be considered as an advantage to exclude any effect on the MWM results rather than memory dysfunction. Treatment of D-gal-treated rats with either Met or Saxa significantly ameliorated the performance of the animals in MWM.

D-gal model is known to produce aging via producing oxidative stress in the brain of experimental animals with symptoms that are particularly related to AD [29]. Correspondingly, the findings of the current study revealed induction of oxidative stress in the brains of rats injected with D-gal as evidenced by the altered MDA and GSH contents, together with the increased level of NOx. Oxidative stress is an important hallmark for both AD and insulin resistance, and it contributes largely to their pathology [34].

Administration of Met and Saxa regained the normal balance of oxidative state in rats with D-gal-induced AD. The antioxidant properties of Met and Saxa have been previously reported [35–38].

The present study also demonstrated the induction of inflammation in brain tissues of rats treated with D-gal as shown by the increased TNF-α content. Inflammation is a key pathogenic factor in the development of AD [39]. Elevated concentration of TNF-α in the brains and cerebro-spinal fluid of AD patients has been reported [40]. The current treatment of rats with Met reduced the inflammation induced by D-gal. The anti-inflammatory property of Met has been shown by other research groups [35, 36]. Saxa also reduced this inflammation, but to a lesser extent as compared to Met.

A state of insulin resistance has been recognized in rats subjected to D-gal shown by the increased level of HbA1c. The induction of insulin resistance in the brain has been supported by the elevated brain contents of insulin and RAGE, as well as the decreased level of insulin receptors [41]. RAGE are overexpressed in response to increased levels of sugars that lead to glycation of the surrounding proteins and formation of advanced glycated end-products (AGE). Numerous in vitro studies showed that these receptors play important roles in mediating the harmful effects of Aβ, which is one of the major hallmarks of AD [42, 43].

Remarkably, this observed insulin resistance could explain the oxidative stress and inflammation that were induced in the brain by D-gal. Insulin resistance reduces glucose utilization and can lead to mitochondrial dysfunction with increased release of free radicals [44, 45]. Moreover, several studies have shown an association between insulin resistance with elevated TNF-α level [46].

Our study showed that the treatment with either Met or Saxa reversed this state of insulin resistance and reduced the accumulation of RAGE, which could explain the enhanced performance of animals in MWM and the improvement of the oxidative and inflammatory grades due to these insulin-sensitizing effects. Thus, the superiority of Met, as compared to Saxa, in protection against D-gal-induced alterations in this study could be explained by the different mechanisms of action of both drugs. Met directly strengthens insulin signaling by modulating adenosine monophosphate kinase activation protein [47, 48], whereas, Saxa increases the incretins levels with consequent inhibition of glucagon release that results in increased insulin secretion, and finally enhanced insulin sensitivity. Therefore, Met still stands as the golden standard as a monotherapy for the treatment of metabolic disorders [47].

To understand the effects of aging and dementia on neurotransmitter levels and how that is related to insulin resistance, we investigated the effects of long-term administration of D-gal on brain levels of acetylcholine and glutamate, which are strongly linked to memory function and severely reduced in AD patients. The results revealed a significant decrease in the levels of both neurotransmitters. Disruption of insulin signaling reduces the level of acetyl-CoA, a precursor of acetylcholine, leading to reduced synthesis of acetylcholine and memory impairment [6]. Similarly, reduced glucose utilization in insulin resistance states results in reduced levels of alpha ketoglutarate which can be considered as a precursor of the excitatory amino acid glutamate and hence reduced glutamate level [49]. Moreover, the deleterious effects of insulin resistance on the survival of neurons can affect cholinergic and glutamatergic neuronal populations, and consequently reduced levels of acetylcholine and glutamate. The treatment with either Met or Saxa in the current study regained normal levels of both neurotransmitters, probably via alleviating the underlying pathology of insulin resistance and impaired insulin signaling.

D-gal model of aging increased the level of phosphorylated tau in the brains of aged animals. This can be attributed to the impaired insulin signaling in neurons that result in tau hyper-phosphorylation and formation of abnormal insoluble tau proteins [3]. Accumulation of tau proteins leads to the formation of neurofibrillary tangles and neuronal death [50]. In line with this, our histopathological findings revealed a formation of Aβ plaques and neurofibrillary tangles in the striatum of D-gal-treated rats with observed neuronal death and degeneration in the hippocampus and cerebral cortex.

Moreover, prominent astrogliosis was revealed in GFAP-immunohistochemically stained sections in which reactive hypertrophied astrocytes were mainly demonstrated around amyloid plaque. These astrocytes play a vital role in degradation of amyloid plaques through the astrocytic processes which internalize and degrade Aβ deposits [51]. However, they secrete inflammatory mediators that lead to neuronal injury [52].

Met or Saxa administration regained the normal levels of both proteins and reversed the normal histology of the brain. Nonetheless, other studies pointed out that Met could increase Aβ expression in neuronal cultures by promoting β- and γ-secretase-mediated cleavage of Aβ protein precursor [11]. Other *in vitro* studies showed that Met can restore abnormal Aβ transport across the blood brain barrier and enhance memory impairment [9]. However, *in vivo* studies provide a more wholesome picture and although Met may increase Aβ production in culture, it can also improve its trafficking and prevent its accumulation; the net result is an improvement in neuronal survival and memory functions.

## Conclusion

Our findings suggest that D-gal model of aging resulted in impairment of learning and memory function in rats by producing a state of impaired insulin signaling and oxidative stress that resulted in a cascade of deleterious events like tau hyper-phosphorylation, β-amyloid accumulation, and inflammation. The use of insulin-sensitizing antidiabetic drugs such as Met and Saxa successfully reversed all these harmful effects in the rats. This suggests their role in alleviating the underlying pathology of insulin resistance and hence, a protection against all the consequence harmful mechanisms.
